# Evidence of compensatory neural hyperactivity in a subgroup of breast cancer survivors treated with chemotherapy and its association with brain aging

**DOI:** 10.3389/fnagi.2024.1421703

**Published:** 2024-12-11

**Authors:** Michele M. Mulholland, Alexa Stuifbergen, Alexa De La Torre Schutz, Oscar Y. Franco Rocha, Douglas W. Blayney, Shelli R. Kesler

**Affiliations:** ^1^Department of Comparative Medicine, The University of Texas MD Anderson Cancer Center, Bastrop, TX, United States; ^2^Division of Adult Health, School of Nursing, University of Texas at Austin, Austin, TX, United States; ^3^Department of Medical Oncology, Stanford University School of Medicine, Palo Alto, CA, United States

**Keywords:** fMRI, breast cancer, brain aging, neural hyperactivity, cognition

## Abstract

**Introduction:**

Chemotherapy-related cognitive impairment (CRCI) remains poorly understood in terms of the mechanisms of cognitive decline. Neural hyperactivity has been reported on average in cancer survivors, but it is unclear which patients demonstrate this neurophenotype, limiting precision medicine in this population.

**Methods:**

We evaluated a retrospective sample of 80 breast cancer survivors and 80 non-cancer controls, aged 35–73, for which we had previously identified and validated three data-driven, biological subgroups (biotypes) of CRCI. We measured neural activity using the z-normalized percent amplitude of fluctuation from resting-state functional magnetic resonance imaging (MRI). We tested established, quantitative criteria to determine whether hyperactivity can accurately be considered compensatory. We also calculated the brain age gap by applying a previously validated algorithm to anatomic MRI.

**Results:**

We found that neural activity differed across the three CRCI biotypes and controls (*F* = 13.5, *p* < 0.001), with Biotype 2 demonstrating significant hyperactivity compared to the other groups (*p* < 0.004, corrected), primarily in prefrontal regions. Alternatively, Biotypes 1 and 3 demonstrated significant hypoactivity (*p* < 0.02, corrected). Hyperactivity in Biotype 2 met several of the criteria to be considered compensatory. However, we also found a positive relationship between neural activity and the brain age gap in these patients (r = 0.45, *p* = 0.042).

**Discussion:**

Our results indicated that neural hyperactivity is specific to a subgroup of breast cancer survivors and, while it seems to support preserved cognitive function, it could also increase the risk of accelerated brain aging. These findings could inform future neuromodulatory interventions with respect to the risks and benefits of upregulation or downregulation of neural activity.

## Introduction

Chemotherapy-related cognitive impairment (CRCI) is experienced by many patients during and after cancer treatment. Despite affecting up to 85% of cancer survivors (Hodgson et al., 2013), CRCI is still poorly understood. Clinical and preclinical research from our group and others suggests that breast cancer chemotherapy upregulates neural activity (Manchon et al., 2016; Chen et al., 2020; Kesler et al., 2009; Ferguson et al., 2007; McDonald et al., 2012; Menning et al., 2017). Although hypoactivity compared to non-cancer controls has also been observed (Pomykala et al., 2013; Saward et al., 2022), hyperactivity is more common, especially longitudinally, and is correlated with subjective cognitive function (McDonald et al., 2012; Menning et al., 2017; Zheng et al., 2020; Chen et al., 2019). Hyperactivity is not limited to breast cancer. For example, Liu et al. found that colorectal cancer patients treated with chemotherapy had greater activation in several brain regions compared to healthy controls. However, it is unknown which patients show neural hyperactivity as most observations have been made by comparing mean activity between patients and controls. A specific subgroup of patients may demonstrate hyperactivity, contributing to the heterogeneity in findings across imaging studies.

To identify CRCI subgroups, we pioneered the application of biotyping to this population (Mulholland et al., 2023; Kesler et al., 2023; Kesler et al., 2020). Specifically, we developed an AI-based algorithm for determining data-driven, latent patterns of brain abnormality (biotypes) in breast cancer survivors. We then examined cognitive phenotypes associated with each biotype (Kesler et al., 2020). As we previously described, Biotype 1 demonstrated impaired cognitive function, Biotype 2 had relatively preserved cognitive function, and Biotype 3 showed moderately impaired cognitive function. Impairment was defined as differing significantly from non-cancer controls, although biotypes also differed significantly from each other. We then cross-validated our biotype algorithm in an independent sample and showed that biotypes had unique demographic, clinical, psychological, and genetic characteristics. In contrast, traditional, symptom-based definitions of cognitive impairment showed no significant differences in these characteristics (Mulholland et al., 2023; Kesler et al., 2020). In the present study, we hypothesized that Biotype 2 would uniquely demonstrate neural hyperactivity given their relatively preserved cognitive function.

**Figure 1:**
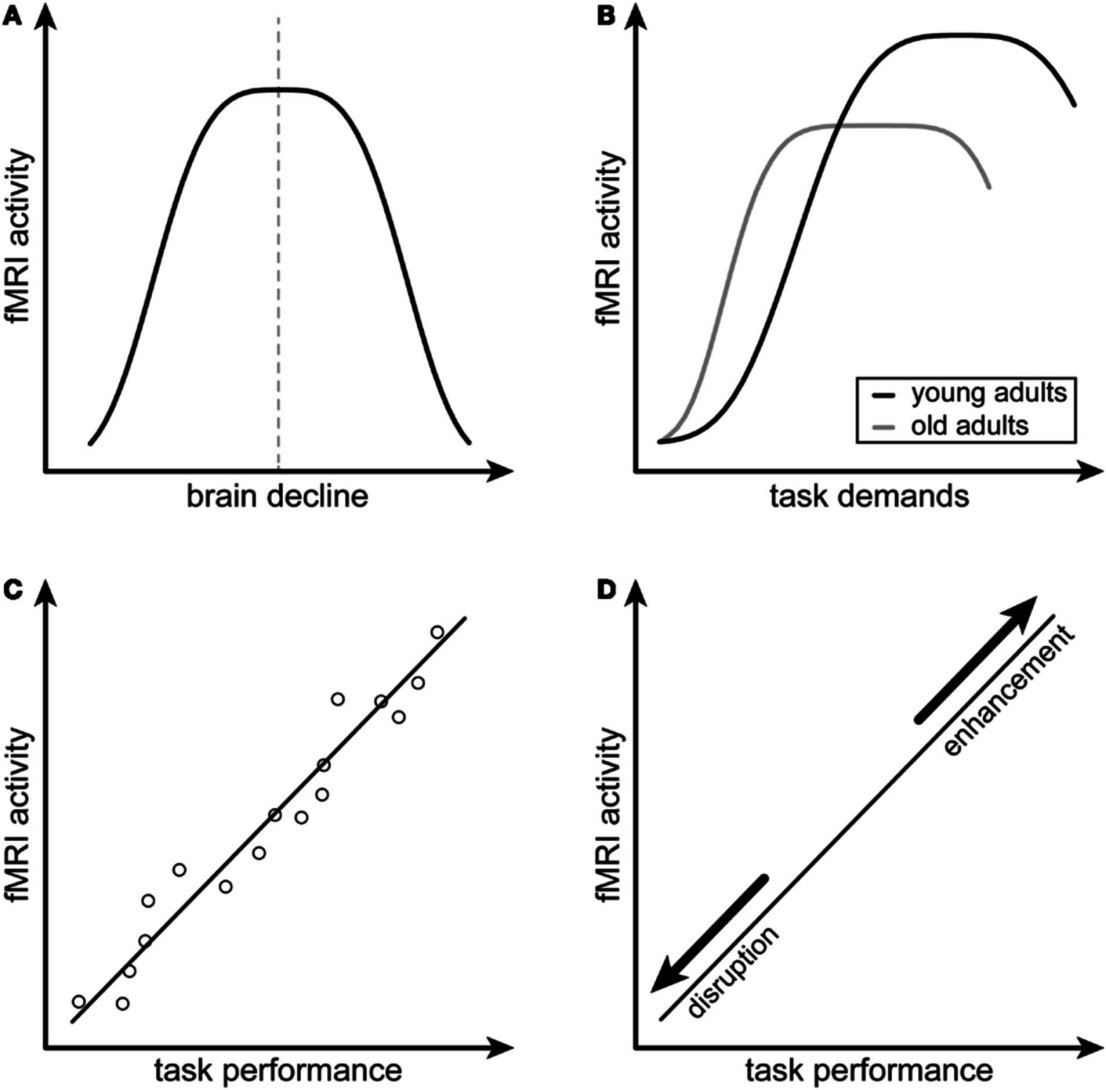
Conceptual model for compensatory neural hyperactivity by Cabeza and Dennis (2013). Criterion **A** indicates that compensatory neural activity as measured by functional magnetic resonance imaging (fMRI), decreases with brain decline. Criterion **B** indicates that compensatory neural activity decreases with increased task difficulty, especially in older individuals. Criterion **C** indicates that compensatory neural activity is positively associated with task performance. Criterion **D** indicates that the relationship between compensatory neural activity and task performance is disrupted or enhanced by modulating the hyperactive brain regions. Figure reprinted from Scheller et al. (2014) under the terms of the Creative Commons Attribution License (CC BY).

The basis for this hypothesis stems from research suggesting that neural hyperactivity may be compensatory or reflect a reorganization of brain function to counteract the decline (Barulli and Stern, 2013; Scheller et al., 2014). In CRCI studies, hyperactivity is often interpreted as compensatory without any evidence to support this claim (Ferguson et al., 2007; Kaiser et al., 2014; Apple et al., 2018; McDonald et al., 2022; Feng et al., 2019). Cabeza and Dennis (2013) proposed four criteria that researchers could use to determine whether brain activity can be attributed to compensation (Figure 1). The first two criteria describe “attempted compensation,” indicating that hyperactivity has an inverted U-shaped relationship with brain decline, task demands, and age. Criterion A indicates that brain activity initially increases in response to brain decline, but as underlying brain structure resources become depleted, brain activity then begins to decline. Criterion B indicates that brain activity increases when a task demands more cognitive processing than the individual has available, but as brain resources are depleted, brain activity again decreases. Cabeza and Dennis (2013) suggest that age affects this relationship; reaching the threshold where resources become depleted occurs earlier in older adults. The remaining two criteria describe “successful compensation,” requiring a positive correlation between hyperactivity and cognitive performance (criterion C) and a change in cognitive performance with alteration of hyperactive regions (criterion D). Criterion D suggests that if we manipulate a hyperactive region (by either disrupting it or enhancing it), we should see a coordinated decline or improvement of the associated compensatory cognitive function (Cabeza and Dennis, 2013).

Hyperactivity may explain the well-known and often controversial discrepancy between elevated subjective cognitive complaints and normal objective cognitive performance in cancer survivors (Kesler et al., 2023). If hyperactivity reflects neural compensation, it could mask the underlying cognitive deficit (Pomykala et al., 2013). However, patient awareness of the additional neural effort required to maintain performance might be reflected in low self-ratings of cognitive function compared to normal or near-normal objective cognitive performance. Therefore, determining if hyperactivity is compensatory would significantly help clarify the inconsistency between objective and subjective CRCI (Hutchinson et al., 2012; O’Farrell et al., 2017) that has frequently resulted in the dismissal of patient reports. Importantly, compensation-related theories suggest methods for enhancing compensation to improve cognition via interventions such as high-frequency repetitive transcranial magnetic stimulation and neurofeedback (Sandrini et al., 2014; Hosseini et al., 2016). Identifying the subgroup of patients who demonstrate hyperactivity may also yield insights regarding modifiable factors that could be applied to other subgroups to help improve cognitive function.

However, compensatory hyperactivity may come at the cost of faster spread of age-related and other neuropathologies making it even more important to identify precisely which patients demonstrate this biotype. Hyperactivity may increase oxidative stress and facilitate the transfer of proteins, such as tau and *α*-synuclein, between neurons, potentially leading to greater accumulation and aggregation (Helwig et al., 2022; Huijbers et al., 2019). With gliomas, hyperactivity and increased functional connectivity may increase the spread of glioma cells and impact patient survival (Krishna et al., 2023). One type of neuropathology often studied in relation to CRCI is accelerated brain aging (Ahles et al., 2012). Brain age is a machine learning-derived neuroimaging measure of brain health, which when compared to chronological age yields the Brain Age Gap (BAG) (Baecker et al., 2021). In our previous studies, we found that while all biotypes had higher brain age than non-cancer controls, Biotype 2 (those with the best cognitive function) had lower brain age than the other biotypes (Kesler et al., 2020; Mulholland et al., 2023). However, we did not measure the more sensitive BAG metric, and it is unknown if neural activity and BAG are related.

To better understand hyperactivity, compensation, and brain aging, we examined neural activity in our CRCI biotypes and tested the compensatory criteria proposed by Cabeza and Dennis (2013). First, we hypothesized that the magnitude of neural activity differs across the CRCI biotypes and controls and is highest in Biotype 2. For compensatory criterion A, we hypothesized that hyperactivity would be related to brain decline, specifically that there would be an inverted-U relationship between gray matter volume and neural activity. For compensatory criterion B, we predicted there would be a significant negative relationship between neural activity and age, with older participants showing less compensatory hyperactivity. For compensatory criterion C, we hypothesized that neural activity would be positively correlated with cognitive performance. Given that this was a retrospective study, testing criterion D was not possible and would require a clinical trial that is beyond the scope of this study. Unrelated to compensatory criteria, we also predicted that higher neural activity would be associated with increased neuropathology (as measured by BAG).

## Methods

### Participants

We evaluated a retrospective sample (data collected between 2008 and 2013) of 80 breast cancer survivors and 80 non-cancer, female controls. The breast cancer survivors were aged between 35 and 73 years and had completed all primary treatments (surgery, radiation, and chemotherapy), excluding hormone blockade, at least 6 months before study enrollment. See Table 1 for participant demographics, such as age, education, and time since treatment. Chemotherapy regimens included doxorubicin/cyclophosphamide (*N* = 3), doxorubicin/cyclophosphamide/ paclitaxel (*N* = 52), doxorubicin/paclitaxel (*N* = 1), doxorubicin/cyclophosphamide/fluorouracil (*N* = 1), doxorubicin/cyclophosphamide/methotrexate (*N* = 5), cyclophosphamide/paclitaxel (*N* = 16), and fluorouracil/epirubicin/cyclophosphamide (*N* = 2). Participants were free from disease and had no history of relapse or recurrence at the time of evaluation. Participants were excluded for neurologic, psychiatric, or medical conditions known to affect cognitive function. The studies involving humans were approved by the Stanford University Institutional Review Board. The studies were conducted in accordance with the local legislation and institutional requirements. The participants provided their written informed consent to participate in this study.

**Table 1:** Participant characteristics.

	Biotype 1 (*N* = 36)	Biotype 2 (*N* = 24)	Biotype 3 (*N* = 20)	Controls (*N* = 80)	Stat	*p*- value
Age in years mean (SD)	49.30 (8.0)	52.52 (6.4)	52.16 (8.7)	49.29 (13.2)	*F* = 0.802	0.494
Education in years mean (SD)	16.39 (2.4)	17.13 (2.7)	16.47 (2.2)	16.99 (2.4)	*F* = 0.782	0.506
Racial/ethnic minority (%)	36%	13%	12%	16%	*X*^2^ = 8.70	0.033
Post-menopause (%)	61%	71%	59%	67%	*X*^2^ = 1.09	0.779
Stage at diagnosis (I, II, III %)	30,65,5%	21,41,38%	18,65,17%		*X*^2^ = 5.42	0.247
Radiotherapy (%)	67%	96%	65%		*X*^2^ = 7.91	0.019
Hormone blockade (%)	61%	75%	64%		*X*^2^ = 1.27	0.531
Months since primary therapy* ended (SD)	26.40 (18.7)	49.67 (33.9)	64.25 (82.1)		*F* = 4.69	0.012

SD, standard deviation. *Surgery, radiation, chemotherapy.

### Neuroimaging data acquisitions

Resting-state fMRI data were collected using a T2*-weighted gradient echo spiral pulse sequence: TR = 2000 ms, TE = 30 ms, flip angle = 80°, and 1 interleave, FOV = 22 cm, matrix = 64 × 64, in-plane resolution = 3.4375 mm2, and number of volumes = 216. A high-resolution, 3D IR-prepared FSPGR anatomic MRI scan was obtained: TR = 8.5, TE = minimum, flip = 15 degrees, TI = 400 ms, BW = +/ − 31.25 kHz, FOV = 22 cm, phase FOV = 0.75, slice thickness = 1.5 mm, 124 slices, 256 × 256 @ 1 NEX, and scan time = 4:33 min. Diffusion tensor imaging data were also collected during this scan session but are not reported here. All sequences were collected using a GE Signa HDx whole-body scanner (GE Medical Systems, Milwaukee, WI).

### Functional brain connectivity

Resting-state fMRI data were preprocessed using Statistical Parametric Mapping 12 (SPM12) (Friston et al., 1994) and CONN 21a (Whitfield-Gabrieli and Nieto-Castanon, 2012) implemented in MATLAB v2023b (MathWorks, Inc., Natick, MA). Briefly, this involved realignment, coregistration with the segmented anatomic volume, spatial normalization, artifact detection (global signal = 3.0 standard deviations, motion = 1.0 mm, rotation = 0.05 mm), band-pass filtering (0.008–0.09 Hz), and correction of non-neuronal noise (Behzadi et al., 2007). Temporal correlations between all possible pairs of 268 regions (Shen et al., 2013) were computed based on the corrected fMRI signal to create a 268×268 functional connectivity matrix for each participant. Thus, the matrix describes the brain network, or connectome, consisting of nodes (regions) and edges (connections).

### Biotypes

We previously developed a machine learning algorithm for determining data-driven, latent patterns of brain abnormality (biotypes) from functional brain connectivity in this cohort. We then examined cognitive phenotypes associated with each biotype based on scores from six tests: Comprehensive Trail Making Tests 1 and 5, Delis–Kaplan Executive Function System Letter Fluency test, Immediate and Delayed Recall from the Rey Auditory Verbal Learning Test, and Global Executive Composite (GEC) of the Behavioral Rating Inventory of Executive Function adult version (Kesler et al., 2020). Biotype 1 demonstrated impaired cognitive function on 6 out of 6 tests, Biotype 2 had relatively preserved cognitive function with impairment on 2 out of 6 tests, and Biotype 3 showed moderately impaired cognitive function with impairment on 4 out of 6 tests. Impairment was defined as differing significantly from non-cancer controls (*p* < 0.05, corrected for multiple comparisons), although biotypes also differed significantly from each other. We then cross-validated our biotype algorithm in an independent sample (Mulholland et al., 2023; Kesler et al., 2020). This study combines both the training (N = 57) and testing (N = 23) samples. See Table 1 for demographic and clinical details of each Biotype.

### Neural activity

We measured neural activity from resting state fMRI using the z-transformed percent amplitude of fluctuation (zPerAF) (Jia et al., 2020). zPerAF is a measure of percent signal change and is calculated for each region as the sum of the absolute values of the standard deviation (z) normalized, mean-centered signal intensities at each time point, divided by the total number of fMRI time points:


$$\frac{1}{n}\;\overset{n}{\underset{i=1}{\sum }}\;|\;\frac{X_{i}-u}{u}\;|\;x\;100\%$$

where $X_{i}$ = signal intensity at the $i^{th}$ time point, u = mean signal across time points, and n = number of time points. zPerAF, as well as mean normalized PerAF (mPerAF), are more reliable than other metrics of resting-state neural activity including ALFF and fALFF (Jia et al., 2020). We chose to utilize zPerAF given our experience that mPerAF can result in infinity values if the mean time series is zero.

### Brain age gap

We estimated brain age from anatomic MRI by utilizing brainageR v2.1, a publicly available algorithm that is one of the most reliable for predicting age from brain MRI (Bacas et al., 2023). The brainageR model was trained on 3,377 healthy individuals (mean age = 40.6 years, SD = 21.4, age range 18–92 years) and tested on an independent dataset of 857 healthy individuals (mean age = 40.1 years, SD = 21.8, age range 18–90 years). The model accepts raw, T1-weighted MRI scans, segments and normalizes them in SPM12 with custom templates, and utilizes the resulting gray, white, and CSF volumes in a Gaussian Processes regression to predict brain age (Cole and Brainage, 2023; Cole et al., 2017). Chronological age was subtracted from estimated brain age to calculate BAG, a metric of brain health wherein a positive BAG represents accelerated brain age (i.e., neuropathology), and a negative BAG represents decelerated brain age (Baecker et al., 2021).

### Statistical analysis

To test the hypothesis that the magnitude of neural activity differs significantly among biotypes, we compared zPerAF between groups (biotypes and controls) using ANOVA with false discovery rate (FDR) correction for multiple comparisons. We also examined Dennis and Cabeza’s compensation criterion (Figure 1). For criterion A (inverted U-shaped relationship between fMRI activity and brain structure), we first fit five different polynomial regression models with zPerAF as a function of gray matter volume, polynomial degrees *h* = 1 to 5, and k-fold cross-validation (*k* = 4-folds) to calculate the test mean squared error (MSE) for each model. We then compared the polynomial model with the lowest MSE to a linear model for goodness of fit using ANOVA and plotted the model to visualize the relationship. Gray matter volume was extracted from anatomic MRI using voxel-based morphometry in SPM12 (Kurth et al., 2015).

We did not have fMRI task data to test compensation criterion B (compensatory hyperactivity decreases with increased task difficulty), but this criterion also indicates that older individuals show reduced compensatory hyperactivity. Therefore, we examined Pearson’s correlation between zPerAF and age. To examine compensation criterion C (positive correlation between fMRI activity and task performance), we conducted Pearson’s correlations between zPerAF and cognitive testing scores. We did not have data to test compensation criterion D (disruption/enhancement of hyperactive brain regions alters the relationship between neural activity and task performance). To test our hypothesis that higher neural activity is associated with higher neuropathology, we conducted a Pearson’s correlation between zPerAF and BAG. Pearson’s correlation *p*-values were FDR-corrected.

## Results

### Neural activity between groups

**Figure 2:**
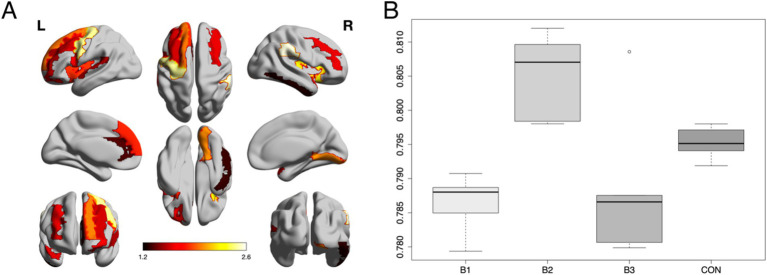
Group differences in neural activity. **(A)** Z-normalized percent amplitude of fluctuation (zPerAF) was significantly different among biotypes and controls (*p* < 0.05, FDR corrected) in the right temporal pole, left anterior cingulate, right inferior temporal gyrus, bilateral insular gyrus, right supramarginal gyrus, left middle frontal gyrus, left superior frontal gyrus, left inferior frontal gyrus, left medial orbital frontal gyrus, left superior medial frontal gyrus, left precentral gyrus, left superior temporal gyrus, right lingual gyrus, and right middle frontal gyrus. The color bar indicates the log of the *p*-value. **(B)** The mean zPerAF across significant regions for each group is displayed as a boxplot. Biotype 2 (B2) showed significant (*p* < 0.004, corrected) hyperactivity compared to the other biotypes and controls. Biotypes 1 and 3 showed significant (*p* < 0.02, corrected) hypoactivity compared to Biotype 2 and controls.

As shown in Figure 2A Z-normalized percent amplitude of fluctuation (zPerAF) was significantly different among biotypes and controls (*p* < 0.05, FDR corrected) in the right temporal pole, left anterior cingulate, right inferior temporal gyrus, bilateral insular gyrus, right supramarginal gyrus, left middle frontal gyrus, left superior frontal gyrus, left inferior frontal gyrus, left medial orbital frontal gyrus, left superior medial frontal gyrus, left precentral gyrus, left superior temporal gyrus, right lingual gyrus, and right middle frontal gyrus. To reduce comparisons, we calculated the mean across these significant regions (Figure 2B) and conducted an ANOVA with Tukey’s HSD *post-hoc* correction (omnibus *F* = 13.5, *p* < 0.001), which indicated that Biotype 2 (*n* = 24) showed significant hyperactivity compared to the other biotypes and controls (*n* = 80; *p* < 0.004, corrected). Biotypes 1 (*n* = 36) and 3 (*n* = 20) showed significant hypoactivity compared to Biotype 2 and controls (*p* < 0.02, corrected), but were not different from each other (*p* = 0.931, corrected). Specifically, 71% of regions were significantly hyperactive in Biotype 2, while 67% were hypoactive in Biotype 1 and 63% were hypoactive in Biotype 3.

### Compensation criterion A

**Figure 3:**
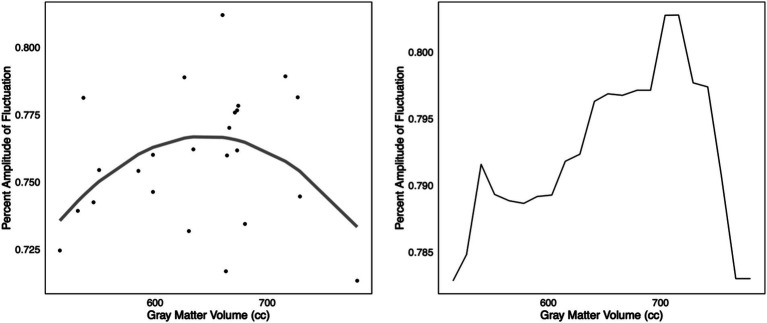
Compensation criterion A. Left: In Biotype 2, k-fold cross-validation indicated that a second-degree polynomial model was the best fit for the relationship between z-normalized percent amplitude of fluctuation and gray matter volumes (adjusted *R*^2^ = 0.14, *p* = 0.013). A scatterplot of this model suggested a potentially inverted, U-shaped relationship, consistent with Dennis and Cabeza’s criterion A for compensatory neural hyperactivity. Right: The relationship was also noted via supplementary k-fold cross-validated random forest regression (adjusted *R*^2^ = 0.35, *p* = 0.010). However, this was in a small sample that requires further validation.

Given that only Biotype 2 showed hyperactivity, compensation criterion analyses were performed only in this group. We used the mean zPerAF across significant regions to reduce comparisons in this small sample. K-fold cross-validation indicated that a second-degree polynomial was associated with the lowest MSE (*X* = 0.0007, *X*^2^ = 0.0006, *X*^3^ = 0.0007, *X*^4^ = 0.0016, *X*^5^ = 0.0031). The second-degree polynomial fit was significant (adjusted *R*^2^ = 0.14, *F* = 4.8, *p* = 0.013) including the polynomial term (*p* = 0.004). The linear fit was not significant (adjusted *R*^2^ = 0.008, *p* = 0.440) and was a significantly poorer fit of the data compared to the second-degree polynomial model (*F* = 9.0, *p* = 0.004). The scatterplot of this model suggested the expected inverted U-shaped relationship (Figure 3). Given the low explained variance, we supplemented the polynomial fit with a random forest regression model, and 4-fold cross-validation (mtry = 2, ntrees = 500), given the superiority of random forest models for fitting complex data (Breiman, 2001). The final model was significant (adjusted *R*^2^ = 0.35, *F* = 5.7, *p* = 0.010). The model plot also suggested an inverted U-shaped relationship (Figure 3).

### Compensation criterion B (partial)

Again, without task fMRI data, we were only able to examine the part of this criterion related to age. We observed the expected negative relationship between age and mean zPerAF across significant regions in Biotype 2 (*r* = −0.49, *p* = 0.020, FDR corrected).

### Compensation criterion C

To reduce comparisons, we used the mean zPerAF across significant regions and evaluated only the cognitive tests that we previously showed to be significantly different in Biotype 2 compared to the other biotypes or controls (Kesler et al., 2020). Consistent with criterion C, members of Biotype 2 showed significant positive correlations between Letter Fluency test score and zPerAF (*r* = 0.60, *p* = 0.008, FDR corrected) as well as GEC and zPerAF (*r* = 0.56, *p* = 0.010, FDR corrected). Both correlations indicated better cognitive function with higher neural activity.

### Neural activity and brain age gap

Although we previously found that Biotype 2 had the lowest brain age of the biotypes (Mulholland et al., 2023), the correlation results between mean zPerAF and BAG indicate that members of Biotype 2 with the highest neural activity have accelerated brain aging (*r* = 0.45, *p* = 0.042, FDR corrected). BAG and zPerAF were not significantly correlated in the other Biotypes (*p* > 0.43, uncorrected).

## Discussion

In this study, we found that neural activity differed across our three CRCI Biotypes and healthy controls, primarily in the prefrontal cortex. The regions included the right temporal pole, left anterior cingulate, right inferior temporal gyrus, bilateral insular gyrus, right supramarginal gyrus, left middle frontal gyrus, left superior frontal gyrus, left inferior frontal gyrus, left medial orbital frontal gyrus, left superior medial frontal gyrus, left precentral gyrus, left superior temporal gyrus, right lingual gyrus, and right middle frontal gyrus. As predicted, Biotype 2 demonstrated significant hyperactivity in these regions compared to the other biotypes and controls.

Further examination of Biotype 2 showed that this hyperactivity met several of the criteria to be considered compensatory (Cabeza and Dennis, 2013). Regarding criterion A, there was a significant, second-degree polynomial relationship between zPerAF and gray matter volume. Second-degree polynomials are U-shaped by definition, and visualization of the model suggested an inverted U-shaped relationship. This finding was supplemented by a random forest regression model. However, given the small sample size and the presence of outliers, further investigation of Criterion A is required. Although we demonstrated robust accuracy and validity of the models via cross-validation, alternative non-linear models may still apply, and potential overfitting remains a concern. Furthermore, gray matter volume may not fully capture structural brain decline. Longitudinal studies that examine the parallel changes in brain structure and function in Biotype 2 patients are necessary to fully test Criterion A.

Although we did not have a measure of task demand to fully test criterion B, we found the expected negative relationship between age and neural activity, with activity decreasing with older age. Next, for criterion C, we found a positive relationship between neural activity and cognitive performance, with hyperactivity being associated with better performance on an objective cognitive test as well as with higher self-ratings of cognitive function. This provides evidence of successful compensatory brain activity but also suggests that patients may not be aware of the additional neural effort required to preserve their cognitive function as we expected. Self-assessment of cognitive function may need to occur closer to objective cognitive loading tasks to evaluate this relationship more precisely. As noted above, we were unable to test criterion D. This would require a behavioral or pharmacologic trial to examine the mediating effect of disruption/enhancement of hyperactive brain regions on the relationship between neural activity and task performance.

This was the first study on CRCI to identify patients exhibiting neural hyperactivity. Identifying which subgroup of patients demonstrates a specific disease biomarker is essential for precision medicine, given that different subgroups will likely have different responses to various interventions. Our results reveal a specific mechanism (prefrontal activity) that may result in CRCI in different groups of patients, which could help determine which treatments and prevention strategies will be most effective for each patient. As we reported previously, there were no distinguishing demographic or clinical characteristics of Biotype 2 expression that could explain their relatively preserved cognitive function (Mulholland et al., 2023; Kesler et al., 2020). Our present results suggest that prefrontal hyperactivity may be responsible for this difference in outcome compared to other patients.

Accordingly, our study was also the first to explicitly test that neural hyperactivity meets the criteria to be considered compensatory (Bernstein et al., 2021; Reuter-Lorenz and Cimprich, 2013; Simó et al., 2013). Our findings are in line with what has been reported as compensatory activity during aging (Cabeza and Dennis, 2013). This is relevant because many studies show that CRCI may reflect age acceleration (Henderson et al., 2014; Wang et al., 2021; Cupit-Link et al., 2017; Hill et al., 2020; Hurria et al., 2016; Sanoff et al., 2014). Cabeza and Dennis (2013) showed that age-related increases in functional connectivity met three of the four compensatory criteria: (A) increased functional activity in the frontal cortex during healthy aging and mild cognitive impairment but decreased functional activity during more severe impairments; (B) examinations of memory load showed that frontal cortex connectivity has an inverted-U relationship with task demand; and (C) age-related increases in frontal cortex functional connectivity was related to successful cognitive performance. Other studies showed similar relationships between age and brain activity as well as brain activity and cognitive performance and task demand (Cabeza et al., 2002; Daffner et al., 2011; Eyler et al., 2011; Riis et al., 2008; Vallesi et al., 2011; Scheller et al., 2018). High-performing older adults (demonstrating preserved cognitive function, similar to CRCI Biotype 2) showed increased frontal cortex activity, compared to low-performing older adults (demonstrating impaired cognitive function, similar to CRCI Biotype 1) (Cabeza et al., 2002; Daffner et al., 2011; Eyler et al., 2011; Riis et al., 2008).

Traumatic life events, including cancer, may accelerate compensatory hyperactivity mechanisms (Persson et al., 2006). However, it remains unclear why or how only Biotype 2 patients demonstrate compensatory neural hyperactivity. Psychological distress, demographic characteristics, or other factors may result in increased neural activity. However, the biotypes were matched for demographics other than higher frequency of ethnic minority status in Biotype 1. Additionally, our previous studies indicated that only Biotype 1 demonstrated significant psychological distress (Mulholland et al., 2023; Kesler et al., 2020). Furthermore, *post-hoc* correlation analysis indicated no significant relationship between mean zPerAF and psychological distress in Biotype 2 (*r* = −0.138, *p* = 0.61) as measured using the Clinical Assessment of Depression (Aghakhani and Chan, 2007). Biotype 2 had a higher frequency of radiation therapy, but at 96%, there was insufficient variance to examine this effect on zPerAF. However, across all three biotypes, there was no *post-hoc* difference in zPerAF between those who did or did not receive radiation therapy (*t* = 0.695, *p* = 0.49). Given the retrospective nature of our studies, we likely lack the data necessary to determine what sets Biotype 2 apart from other patients. It will be essential to conduct prospective biotyping studies to determine whether there are modifiable factors contributing to the cognitive phenotype of Biotype 2.

However, while compensatory hyperactivity in Biotype 2 may help explain their increased cognitive resilience, they could also be at risk for accelerated brain aging. Our results showed a positive relationship between neural activity and brain age gap (BAG, a proxy of neuropathology). In a study of healthy adults, Scheller et al. (2018) found that APOE variant and brain age moderated the relationship between neural hyperactivity and cognitive performance. Specifically, APOEe4 carriers with higher brain ages had increased frontal cortex activity which correlated with preserved cognitive function (Scheller et al., 2018). Unfortunately, we cannot determine the directional nature of this relationship in either study, as data were collected at a single time point. Neurodegeneration may result in hyperactivity, or this relationship could be bidirectional. In the current study, given that (1) hyperactivity was observed only in Biotype 2 and met several criteria for being compensatory and, (2) these patients demonstrated a unique relationship between BAG and hyperactivity while simultaneously having the lowest BAG, it is more likely that hyperactivity in this subgroup results in neurodegeneration rather than the reverse. However, further studies are required to better evaluate these relationships.

Previous studies of cognitive impairment in aging adults found a relationship between accelerated brain aging, worsening cognitive function, and clinical disease severity (Franke and Gaser, 2019; Gaser et al., 2013; Franke and Gaser, 2012). Brain age at baseline predicted a future advancement from mild cognitive impairment to Alzheimer’s disease 3 years later, and these data were used to create hazard ratios for the development of Alzheimer’s based on brain age (Franke and Gaser, 2019; Gaser et al., 2013; Franke and Gaser, 2012). Future studies should include repeated brain imaging and cognitive testing for cancer survivors to determine whether compensatory activity precedes increases in brain age, or whether brain age can predict further future cognitive declines in those with CRCI.

Our results provide novel insights regarding potential interventions for CRCI by identifying *who* has hyperactivity and *where* hyperactivity occurs. Methods for enhancing compensation to improve cognition include neuromodulation (Barulli and Stern, 2013; Scheller et al., 2014). Neuromodulation is a strong candidate for addressing abnormal neural activity as it is already FDA-approved for use in other neuropsychiatric conditions (Johnson et al., 2013). Future prospective studies of neural hyperactivity could determine which patients might benefit most from such strategies, including some of the potential risks (brain aging) and benefits (compensatory cognition) of upregulation versus downregulation, respectively.

This study is not without limitations. Sample sizes for each biotype were relatively small. We addressed this by reducing comparisons when possible, in combination with multiple comparisons correction, but further study in larger samples is required. As mentioned previously, as this was a retrospective study, we did not have a measure of task demand to be able to fully test Criterion B. Future prospective studies should include measures of task demand when studying CRCI; for example, dual or concurrent tasks, tasks that vary demand, linguistic analyses, physiological measures, or self-report measures such as the NASA Task Load Index (Hart and Staveland, 1988; Sunderaraman et al., 2013; Vizer and Sears, 2017; Scholey et al., 2001; Révész et al., 2016; Fleming et al., 2023; Charles and Nixon, 2019; Ayres et al., 2021). Including self-report measures of cognitive load or demand after each objective cognitive test could also assess whether patients are aware of any increased neural effort associated with their performance.

In addition, interventions that target the hyperactive brain regions could be examined to directly test criterion D. For example, researchers could utilize methods of brain stimulation (e.g., transcranial magnetic stimulation, transcranial alternating current stimulation, transcranial direct current stimulation) or neurofeedback with CRCI patients to examine the effect of these non-invasive brain manipulations on cognition (e.g., Vosskuhl et al., 2018; Kehler et al., 2020; Klink et al., 2020; Kuo and Nitsche, 2012; Laborda-Sánchez and Cansino, 2021; Loriette et al., 2021; Trambaiolli et al., 2021). Animal models could be used to examine the effects of direct electrical stimulation of brain regions linked to CRCI. Both BAG and zPerAF are measured from neuroimaging and although they are derived from different imaging modalities, there is inherent neurobiological overlap. Therefore, future studies should examine the effect of neural activity on non-imaging biomarkers of neurodegeneration such as peripheral tau and amyloid-beta (Henneghan et al., 2020), for example. Alternatively, BAG derived from fMRI may yield different results. However, validated, publicly available BAG algorithms currently utilize anatomic MRI rather than fMRI. Our study used a between-subjects approach to evaluating Criterion C, but comparing the performance of the same individual across different trials (within-subjects) may provide further insights regarding neural response to correct versus incorrect performance. However, the variability in hyperactivity between individuals complicates this relationship, and therefore, mixed evidence from between-and within-subjects data is needed. We defined our biotypes using functional connectivity and measured neural activity using perAF, which are both derived from resting state fMRI. However, our biotypes were cross-validated, and perAF was not significantly different in the training and testing samples (*p* > 0.315), suggesting that our findings generalize well across independent data. While there may be some correlation between functional connectivity and perAF, this does not imply redundancy as these metrics capture distinct neurophysiological characteristics. However, future studies with alternative metrics of brain function are required. Finally, this study only includes breast cancer survivors and those who have undergone treatment for other cancer types with different treatment regimens may differ in brain activity post-treatment. They may not display the same compensatory mechanisms.

The current study demonstrates that the neural hyperactivity observed in CRCI Biotype 2 potentially meets most of the compensatory criteria. This neural compensation may explain the preserved cognitive function observed in Biotype 2 compared to the other CRCI Biotypes. Furthermore, neural hyperactivity may be related to accelerated brain aging. Future studies should include measures of cognitive decline and manipulation of frontal cortex activity to further test the compensatory criteria, as well as collect longitudinal data to better elucidate the relationship between hyperactivity and brain aging.

## Data Availability

The data analyzed in this study is subject to the following licenses/restrictions: All data relevant to the study are included in the article. The original MRI data underlying this article cannot be shared publicly due to data protection regulation. Requests to access these datasets should be directed to srkesler@austin.utexas.edu.
